# Association of circulating vaspin levels and patients with metabolic-associated fatty liver disease: a systematic review and meta-analysis

**DOI:** 10.1186/s12944-022-01658-2

**Published:** 2022-07-02

**Authors:** Yuqing Zhu, Yani Ke, Yijie Hu, Kaihan Wu, Shan Liu, Jie Hu

**Affiliations:** 1 The First Clinical Medical College of Zhejiang Chinese Medical University, No 548, Binwen Road, Hangzhou, 310051 Zhejiang Province China; 2 The Second Clinical Medical College of Zhejiang Chinese Medical University, No 548, Binwen Road, Hangzhou, 310051 Zhejiang Province China; 3 The Third Clinical Medical College of Zhejiang Chinese Medical University, No 548, Binwen Road, Hangzhou, 310051 Zhejiang Province China; 4 Department of Clinical Evaluation Center, The First Affiliated Hospital of Zhejiang Chinese Medical University, No. 54, Youdian Road, Hangzhou, 310006 Zhejiang Province China; 5 Department of Infectious Diseases, The First Affiliated Hospital of Zhejiang Chinese Medical University, No. 54, Youdian Road, Hangzhou, 310006 Zhejiang Province China

**Keywords:** Metabolic-associated fatty liver disease, Nonalcoholic fatty liver disease, Nonalcoholic steatohepatitis, Vaspin, Meta-analysis

## Abstract

**Background:**

The incidence rate of metabolic-associated fatty liver disease (MAFLD) is increasing annually; however, there are still no effective methods for establishing an early diagnosis and conducting real-time tracing. Vaspin can affect the metabolic processes in the body, and it is closely associated with many metabolic diseases. Many previous studies have speculated on the association between vaspin and MAFLD, but the results of these studies have not been conclusive. This meta-analysis examined the differences in circulating vaspin levels between patients with MAFLD and healthy individuals.

**Methods:**

Six databases and other sources were searched with free terms and Medical Subject Headings terms, and a total of 13 articles were included (900 cases and 669 controls). RevMan 5.3 and Stata 16 were used for analysis. The standardised mean difference (SMD) and 95% confidence interval (CI) were used to assess the overall outcomes. Cohen’s kappa coefficient was applied to examine the differences between the two authors in the selection of studies and in the evaluation of the quality of evidence for the studies.

**Results:**

The results demonstrated that there was no significant difference in the circulating vaspin levels between the MAFLD group and healthy group (SMD = 0.46, 95% CI: [− 0.12, 1.04]). The subgroup analysis suggested that area and body mass index (BMI) may be the sources of heterogeneity, and the results of univariate meta-regression analysis were consistent with those of the subgroup analysis (*P* = 0.005 and *P* < 0.001, respectively). Furthermore, BMI may better explain the source of heterogeneity (*P* = 0.032) in the multivariate meta-regression analysis.

**Conclusion:**

In summary, no significant correlation was observed between the circulating vaspin levels and MAFLD. BMI may be an important factor affecting this correlation, which may provide a reference for further studies on mechanism and diagnosis of MAFLD.

**Supplementary Information:**

The online version contains supplementary material available at 10.1186/s12944-022-01658-2.

## Introduction

Nonalcoholic fatty liver disease (NAFLD), a common type of chronic hepatic disease, may affect approximately 25–30% [1] adults in the general population, and the related mortality from NAFLD increased rapidly in the recent [2]. Therefore, NAFLD has a place among the main public health problems. Since NAFLD is a multi-organ system disease [3] with a heterogeneous pathogenesis, experts have jointly proposed replacing it with metabolic-associated fatty liver disease (MAFLD) [4]. MAFLD is considered to be a clinicopathological syndrome characterised by hepatic steatosis, and it includes a continuum of hepatic conditions varying in the severity of injury and fibrosis, which is histologically defined as steatosis, inflammation, and ballooning of the hepatocytes [5–7]. The pathogenesis of MAFLD is complex, and it has been established that MAFLD is strongly related to metabolic disorders, such as obesity and type 2 diabetes mellitus (T2DM) [8–10]. The clinical monitoring and screening methods for MAFLD are currently limited and may cause trauma to patients, resulting in delays in the diagnosis of the disease and opportunities for an early intervention. Thus, it would be meaningful to explore more biomarkers that can indicate the occurrence and progression of MAFLD.

Adipokines are peptides [11] produced by adipose tissue with autocrine, paracrine, and endocrine functions that contribute to the development of simple steatosis (SS), nonalcoholic steatohepatitis (NASH), and even cirrhosis [12]. Several researches have brought in focus on the relationship between various adipokines and MAFLD. Polyzos et al. [13] summarised the relationship between leptin and MAFLD, and they concluded that higher circulating leptin levels were observed in MAFLD group than in healthy controls. Hu et al. [14] also observed that irisin is associated with MAFLD in the Asian population. Vaspin, a newly discovered adipokine, is also known as the visceral adipose tissue-derived serine protease inhibitor. It is expressed in visceral and subcutaneous adipose tissues and can also be found in other organs, such as the pancreas, liver, stomach, and skin [15]. Evidence demonstrates that vaspin has correlation with many metabolic diseases, such as obesity, diabetes, and metabolic syndrome, and its level is positively correlated with the risk of many vascular and metabolic dysfunctions [16, 17], so it may be used as a biomarker for MAFLD. Consequently, more studies have focused on the vaspin levels and MAFLD, but the fineness and consistency of the conclusions still need further discussion. This study intended to find the relationship between vaspin and MAFLD to provide more data on establishing an early diagnosis of MAFLD and the possible mechanism of vaspin in the pathogenesis of MAFLD.

## Methods

### Literature search

The protocol for this meta-analysis was published in PROSPERO (registration ID: CRD42022301367). The published protocol is provided in Additional file 1. According to the Preferred Reporting Items for Systematic Reviews and Meta-Analyses (PRISMA) criteria [18] and the recommendations of the Cochrane Collaboration, this study was performed and completed in Additional file 2.

Two researchers in the team searched six databases: Cochrane Library, EMBASE, PubMed, CNKI, CBM, and Wanfang. Additionally, a supplementary retrieval was performed using Google Scholar. The search was performed until 13 February 2022. A combination of subject words, keywords, and free words were used for the retrieval. The following MeSH terms and search words were used: ‘NAFLD’, ‘Non-alcoholic fatty liver disease’, ‘Nonalcoholic fatty liver disease’, ‘NASH’, ‘Non-alcoholic steatohepatitis’, ‘Nonalcoholic steatohepatitis’, ‘MAFLD’, ‘Metabolic associated fatty liver disease’, ‘Fatty liver’, ‘Nonalcoholic fatty liver’, ‘Vaspin’, ‘Vaspin protein’, ‘Serine Proteinase Inhibitors’, ‘Serpins’, ‘SERPINA12 protein, human’, and the specific strategy is detailed (see Additional file 3). There were language limitations set to both English and Chinese. In order to prevent any omission of relevant research, the references cited by the included studies were also assessed roundly. For articles with an unavailable full text or missing data, the corresponding author was contacted to obtain relevant information comprehensively via email.

### Study selection

As for the selection of studies, the whole process was independently conducted by two authors. Any disagreements in the eligibility of studies between reviewers were all resolved through discussion with a third reviewer.

The inclusion criteria must satisfy the followings: (1) the MAFLD group consisted of patients with or without T2DM who met the imaging or histological clinical diagnostic criteria and were clearly diagnosed with MAFLD, NAFLD, SS, nonalcoholic fatty liver (NAFL), or NASH; (2) the control group included only healthy individuals with normal liver ultrasonography and normal liver function test results; (3) both groups included adults (age ≥ 18 years); (4) studies with the outcome of circulating vaspin levels in either serum or plasma; and (5) observational studies, including case-control and cohort studies.

The exclusion criteria consisted of the following: (1) studies on secondary liver fat accumulation, such as hereditary disorders, a history of heavy alcohol consumption, or have taken steatogenic medication, and studies involving patients who had severe organic disease; (2) vaspin levels not derived from the serum or plasma; (3) studies conducted by the same team on the same study cohort, with overlapping datasets, or duplicate publications; (4) studies for which the full-text was unable to be obtained, with missing critical data, or without any relevant data obtained after contacting the authors; and (5) case reports, reviews, protocols, comments, or letters.

### Data extraction and quality assessment

The data extraction was performed by two authors without any communication, and any confusing parts were judged by a third reviewer. The extracted information included: last name of the first author, published year, country, number of each group, basic information of each group (such as age, sex, and body mass index [BMI]), homeostasis model assessment of insulin resistance (HOMA-IR) values, diagnostic methods, vaspin measurement method, and circulating vaspin levels in each study.

The Newcastle–Ottawa scale (NOS) [19] score was utilised to evaluate the qualities of the included studies, which consists of the assessments of the selection, comparability, and exposure. The grading of recommendation, assessment, development, and evaluation (GRADE) approach was performed to assess the certainty of this study (https://gdt.gradepro.org).

Cohen’s kappa coefficient [20] was performed to examine the differences between the two authors in the selection of the studies and in the evaluation of the evidence quality.

### Statistical analysis

RevMan 5.3 and Stata 16 (Stata Corporation, TX, USA) were two software used for analysing and integrating the data. All studies applied the standardised mean difference (SMD) to evaluate the outcomes. Besides, the normally distributed data are expressed as the mean ± standard deviation (SD). To perform this meta-analysis, the *I*^*2*^ test and Q-test of Cochran were used to evaluate the heterogeneity among the included studies. The fixed-effects model was implemented when heterogeneity was low (*P* ≥ 0.1). And the random-effects model was implemented when heterogeneity was high (*P* < 0.1) [21, 22]. Furthermore, subgroup and meta-regression analysis were used to find out the sources of heterogeneity and sensitivity analysis was used to evaluate the stability of the outcomes by the sequential omission of individual studies. In addition, publication bias was evaluated through Egger’s test, Begg’s test, and funnel plots [23].

## Result

### Study selection

**Fig. 1:**
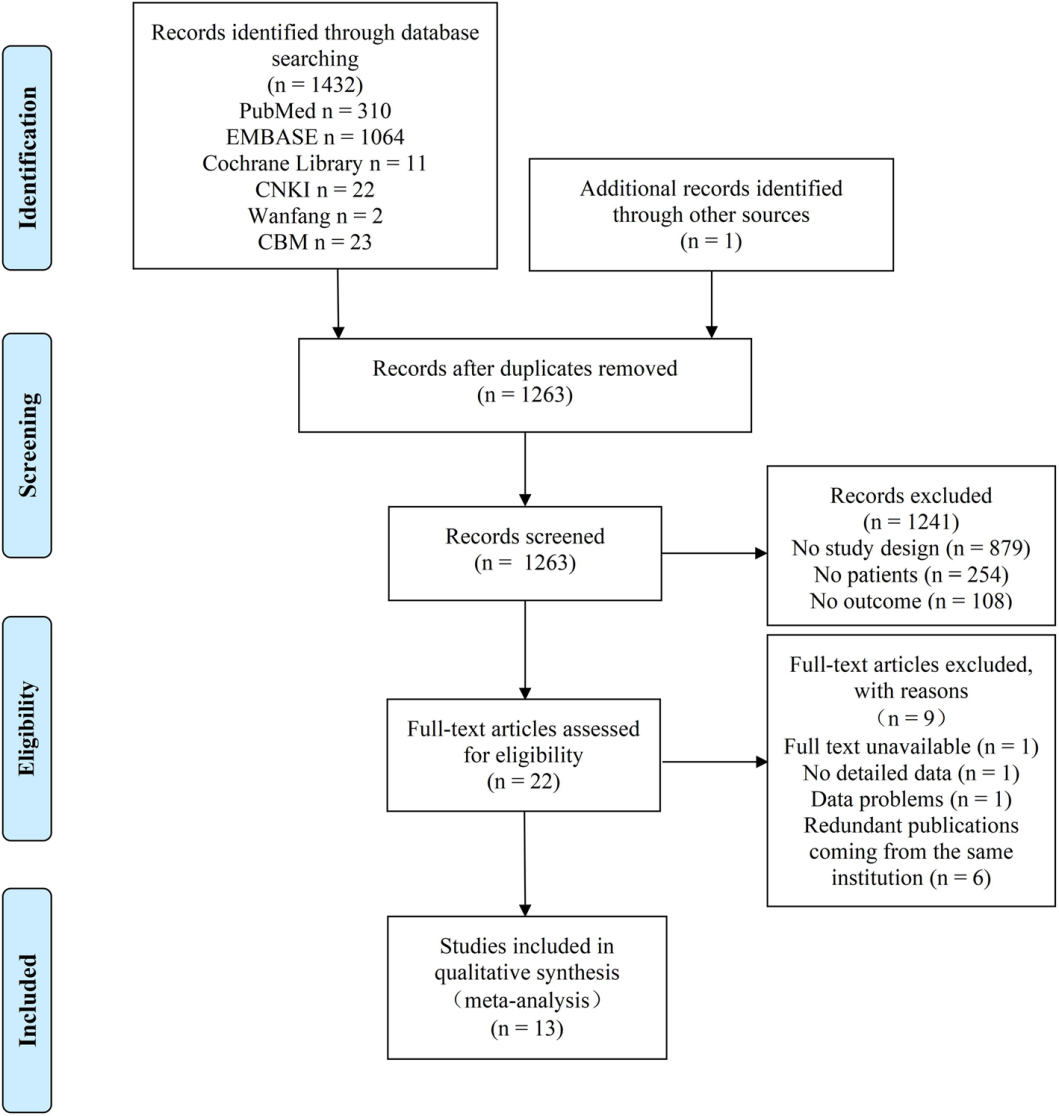
Flowchart of study inclusions and exclusions

The whole process of the study selection is shown in the form of a PRISMA flow diagram (Fig. 1). Six databases were searched by two members simultaneously, and 1432 articles were obtained. In addition, an article was obtained through other sources [24]. Duplicates were removed, and 1263 articles were screened. In the final analysis, there were only 13 articles (900 cases and 669 controls) included. These articles covered three continents and six countries, namely China, Turkey, Poland, Greece, Egypt, and Iran. The patients and healthy people were not limited by sex, and their average median ages ranged from 30 to 69.3. The median BMI of the patients with NAFLD ranged from 21.65 to 34.59 kg/m^2^, and the median BMI of the healthy controls ranged from 22.08 to 30.5 kg/m^2^. Table 1 displayed more information of the included studies in detail.

**Table 1:** Baseline characteristics of the studies included in the meta-analysis

No.	Author	Year	Country		No. of Patients		Sex(F/M)		Age		BMI (kg/m^**2**^)		Detection Method
					NAFLD group	Control group	NAFLD group	Control group	NAFLD group	Control group	NAFLD group	Control group	
1	Aktas [25]	2011	Turkey		91	81	43/48	39/42	47 ± 9	46 ± 11	31.3 ± 5.1	26.5 ± 3.4	ELISA kit (Alpco Diagnostics, Salem, NH, USA)
2	Fu [26]	2019	China		188	100	101/87	53/47	48.26 ± 8.2	48.04 ± 7.2	21.65 ± 2.37	22.08 ± 2.25	ELISA kit (KLANG, Shanghai, China)
3	Genc [27]	2011	Turkey		50	30	50/0	30/0	32 ± 6	30 ± 5	28.5 ± 3.2	23.4 ± 2.9	ELISA kit (Catalog number 44-VASHU, Alpco Immu_x005fnoassays, Salem, NH, USA)
4	Ismail [24]	2019	Egypt	Total	30	15	5/25	2/13	/	41.8 ± 7.11	/	23.86 ± 2.19	ELISA kit (Cat No. In- Hu3180: China Bioneovan Co., Ltd.)
				SS	15		0/15		47.93 ± 4.31		34.59 ± 3.75		
				NASH	15		5/10		50.20 ± 9.10		34.33 ± 4.98		
5	Kukla [28]	2010	Poland		41	20	26/15	10/10	45.7 ± 12.7	40.6 ± 5.5	30.4 ± 3.3	24.4 ± 4.1	ELISA kit (Catalogue No. V0712TP, AdipoGen Inc., Seoul, South Korea)
6	Montazerifar [29]	2017	Iran		41	41	13/28	17/24	39.8 ± 8.9	36.7 ± 8.5	28.3 ± 4.2	25.1 ± 3.6	ELISA kit (Cat No. E2014Hu: Shanghai Crystal Day Biotech Co., Ltd.)
7	Polyzos [30]	2016	Greece	Total	29	25	7/22	5/20	/	53.6 ± 1.8	/	30.5 ± 0.8	ELISA kit (RayBiotech, Norcross, GA, USA)
				SS	15		5/10		53.9 ± 2.6		31.9 ± 1.3		
				NASH	14		2/12		54.8 ± 1.6		33.9 ± 1.6		
8	Su [31]	2021	China		138	129	53/85	50/79	*a	42.7 ± 7.5	27.9 ± 4.6	23.1 ± 3.6	ELISA kit (JingTian, Shanghai, China)
9	Waluga [32]	2019	Poland		25	25	12/13	11/14	31 ± 10	42 ± 15	31.14 ± 6.07	22.15 ± 0.83	ELISA kit (BioVendor – Laboratorni Medicina a.s., Brno, Czech Republic)
10	Xia [33]	2011	China	NAFLD	40	41	22/18	23/18	68 ± 8.8	69.3 ± 9.3	24.93 ± 3.61	23.71 ± 5.34	ELISA kit (ADL, USA)
				NAFLD +T2DM	44		24/20		69.2 ± 9.6		24.71 ± 5.34		
11	Yan [34]	2019	China		86	56	*b	31/25	45.29 ± 7.35	45.32 ± 7.23	23.55 ± 0.27	23.53 ± 0.25	ELISA kit (Aidibo, Beijing, China)
12	Yilmaz [35]	2011	Turkey		54	56	26/28	27/29	47 ± 10	46 ± 11	31.2 ± 4.9	24.9 ± 3.1	ELISA kit (Alpco Diagnostics, Salem, NH, USA)
13	Yu [36]	2012	China		43	50	23/20	24/26	55.42 ± 5.87	56.82 ± 5.22	27.48 ± 4.74	22.73 ± 3.01	ELISA kit (Kangtai, Beijing, China)

*Abbreviations*: *M* Male, *F* Female, *BMI* Body mass index, *NAFLD* Nonalcoholic fatty liver disease, *NASH* Nonalcoholic steatohepatitis, *SS* Simple steatosis, *T2DM* Type 2 diabetes mellitus

Data are presented as the mean and SD or as the count, as appropriate

^*a^. Data on the ages of the NAFLD patients in the table do not agree with the data mentioned in the paragraph of the *Su 2011* study

^*b^. There is an obvious error in the original data pertaining to sex for the NAFLD patients in the *Yan 2019* study

### Quality assessment

The NOS scale was performed for quality evaluation of the study. The average score of NOS in the 13 articles was 5.85 (Table 2). Most of the articles had a NOS score of six or above, while three articles [27, 28, 32] obtained a NOS score of only five due to a lack of representativeness. The results obtained by the GRADE system also suggested that this study had very low reliability of the overall evidence (see Additional file 4). Since all of the studies belong to observational studies, the score could only start from low. Combining many factors (the above NOS score, the high heterogeneity, and the confounding variables among studies), it prompted the result of very low certainty.

**Table 2:** Newcastle-Ottawa Scale (NOS) score of included articles

No.	Author	Year	Selection				Comparability	Exposure			Total	Average
			Adequate definition	Representativeness	Selection of Controls	Definition of Controls		Ascertainment of exposure	Same method	Non-Response rate		
1	Aktas [25]	2011	1	1	1	1	0	1	1	0	6	5.85
2	Fu [26]	2019	1	1	1	1	0	1	1	0	6	
3	Genc [27]	2011	1	0	1	1	0	1	1	0	5	
4	Ismail [24]	2019	1	1	1	1	0	1	1	0	6	
5	Kukla [28]	2010	1	0	1	1	0	1	1	0	5	
6	Montazerifar [29]	2017	1	1	1	1	0	1	1	0	6	
7	Polyzos [30]	2016	1	1	1	1	1	1	1	0	7	
8	Su [31]	2021	1	1	1	1	0	1	1	0	6	
9	Waluga [32]	2019	1	0	1	1	0	1	1	0	5	
10	Xia [33]	2011	1	1	1	1	0	1	1	0	6	
11	Yan [34]	2019	1	1	1	1	0	1	1	0	6	
12	Yilmaz [35]	2011	1	1	1	1	0	1	1	0	6	
13	Yu [36]	2012	1	1	1	1	0	1	1	0	6	

The result of the assessment with Cohen’s kappa in the selecting studies was 0.956, suggesting an almost perfect agreement between the two authors. The Cohen’s kappa statistic for evidence quality evaluation was 0.447, indicating moderate agreement. Therefore, the third author determined the final NOS score.

### Correlation between vaspin levels and MAFLD

**Fig. 2:**
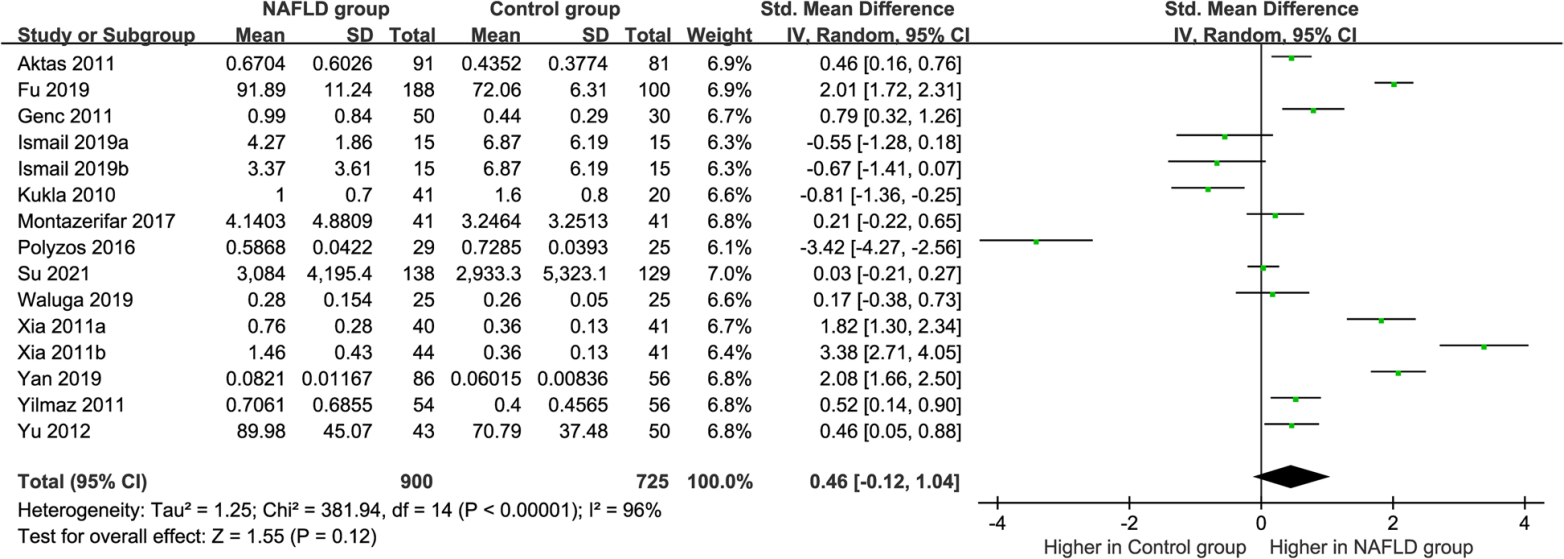
Forest plot of circulating vaspin levels between MAFLD and the healthy control group (Random-Effects Model, SMD)

The pooled meta-analysis of the 13 studies is shown in Fig. 2. High heterogeneity was obvious (*P* < 0.00001, *I*^*2*^ = 96%); therefore, the random-effects model became the first choice. The circulating vaspin levels exhibited no significant difference between the MAFLD and healthy individuals, which had an overall SMD of 0.46 [− 0.12, 1.04].

**Fig. 3:**
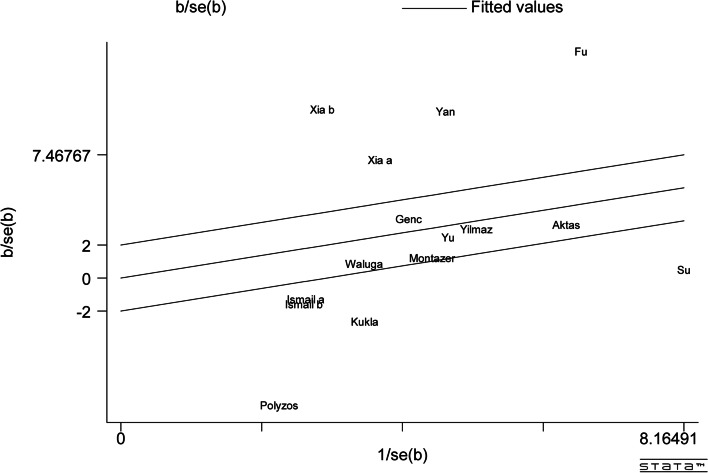
Galbraith Test result of circulating vaspin levels between MAFLD and the healthy control group

Obvious heterogeneity was also observed in the Galbr diagram (Fig. 3). More than half of the studies were not within the reasonable range. Therefore, subgroup and meta-regression analysis were necessary for the exploration of the possible sources of heterogeneity.

### Subgroup analysis by area

**Fig. 4:**
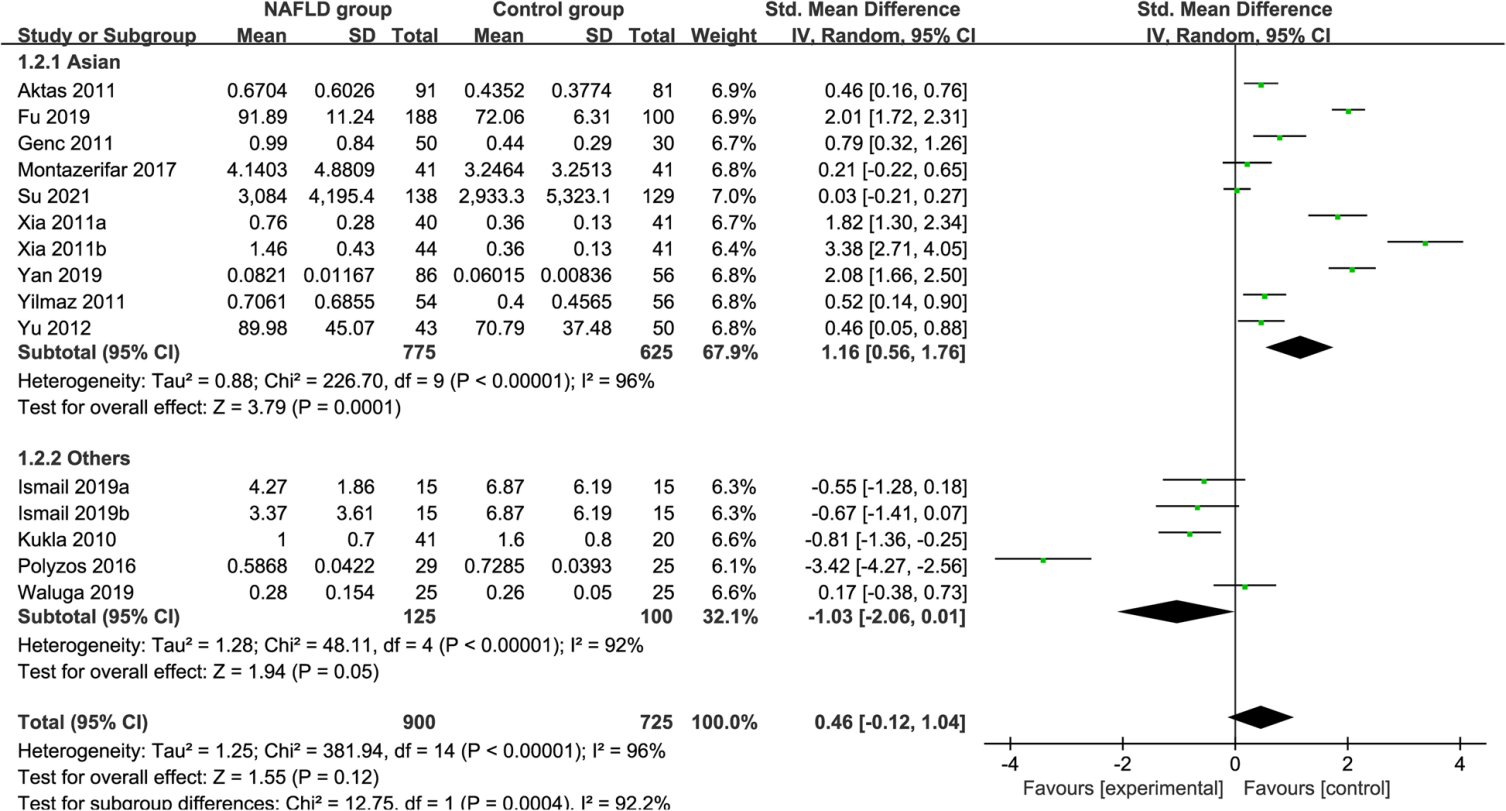
Forest plot of circulating vaspin levels between MAFLD and the healthy control group by area (Random-Effects Model, SMD)

Subgroup analysis divided by the area is shown in Fig. 4. The random-effects model was chosen owing to the high heterogeneity among the studies (Asians: *P* < 0.00001, *I*^*2*^ = 96%; Others: *P* < 0.00001, *I*^*2*^ = 92%). The pooled analysis demonstrated that the circulating vaspin levels of the MAFLD patients showed significant higher than those of the healthy group in the Asian subgroup. In contrast, in the other areas, the circulating vaspin levels exhibited no significant difference between the MAFLD and healthy individuals (Asians: SMD = 1.16 [0.56, 1.76]; others: SMD = − 1.03 [− 2.06, 0.01]). Perhaps area may be the source of heterogeneity, but this requires further verification.

### Subgroup analysis by age

**Fig. 5:**
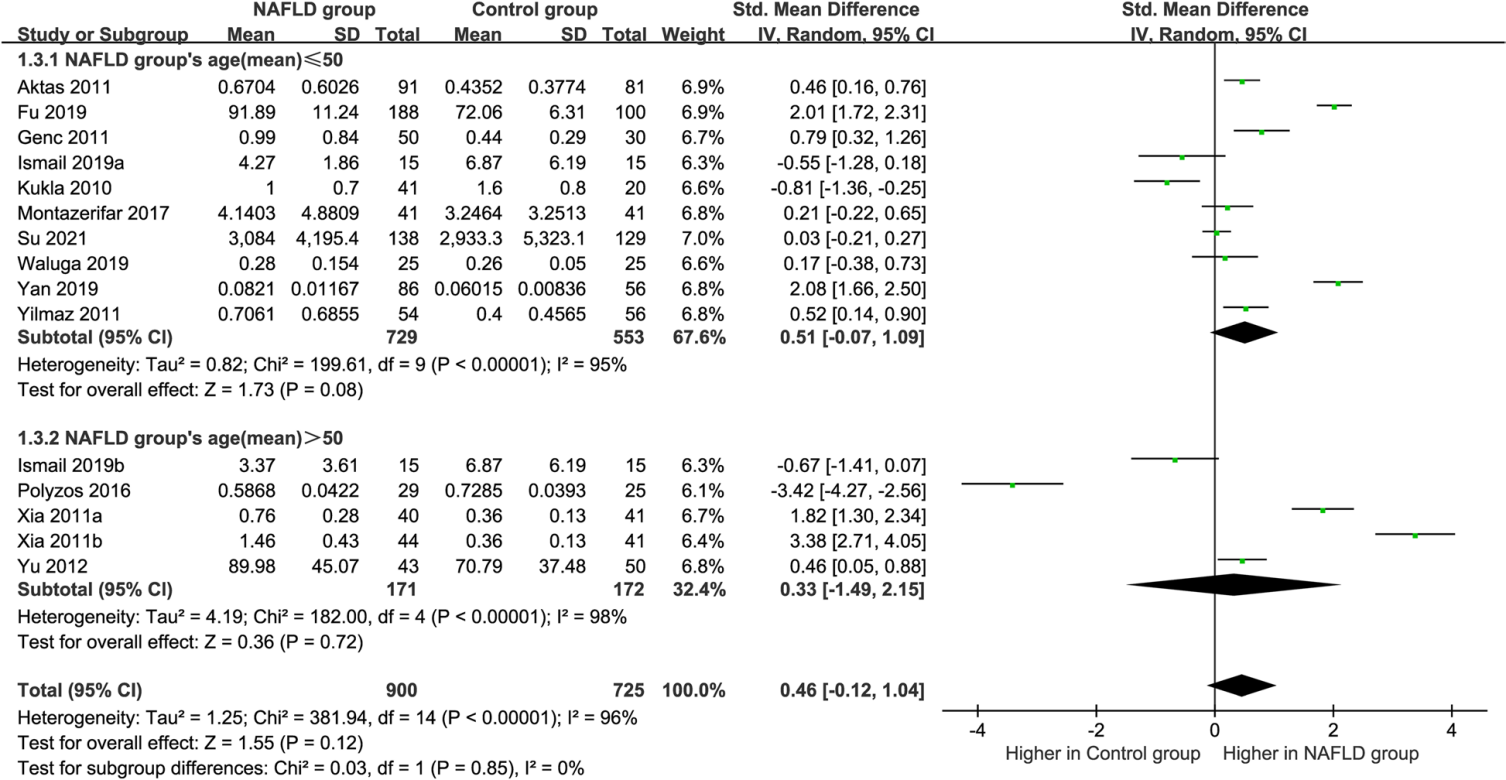
Forest plot of circulating vaspin levels between MAFLD and the healthy control group by age (Random-Effects Model, SMD)

The outcomes of the subgroup analysis divided by age is displayed in Fig. 5. Because of the high heterogeneity (age ≤ 50: *P* < 0.00001, *I*^*2*^ = 95%; age > 50: *P* < 0.00001, *I*^*2*^ = 98%), a random-effects model was selected again. No significant difference was found in the circulating vaspin levels between MAFLD patients and healthy group, regardless of whether the age of MAFLD patients was greater than 50 or less than 50 (age ≤ 50: SMD = 0.51 [− 0.07, 1.09]; age > 50: SMD = 0.33 [− 1.49, 2.15]).

### Subgroup analysis by BMI

**Fig. 6:**
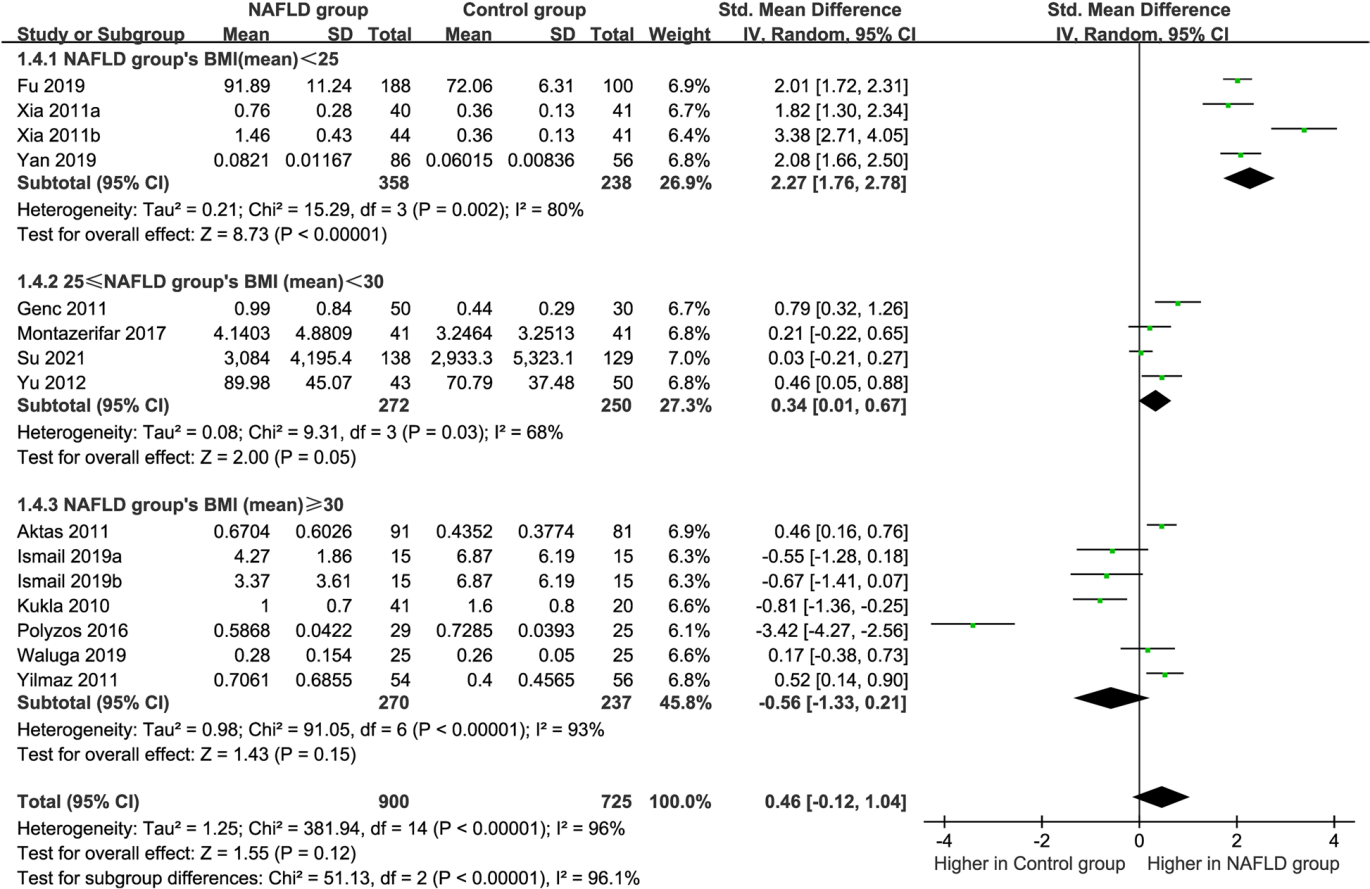
Forest plot of circulating vaspin levels between MAFLD and the healthy control group by BMI (Random-Effects Model, SMD)

Subgroup analysis was implemented in terms of BMI (Fig. 6). The subjects were divided into subgroups according to the criteria of the World Health Organization: a BMI greater than or equal to 25 kg/m^2^ was defined as overweight, and obesity was defined as a BMI greater than or equal to 30 kg/m^2^ [37]. For the MAFLD group with a BMI < 25 kg/m^2^ and 25 kg/m^2^ ≤ BMI < 30 kg/m^2^, the circulating vaspin levels were significantly higher than those in healthy individuals (SMD = 2.27 [1.76, 2.78], *P* = 0.002; SMD = 0.34 [0.01, 0.67], *P* = 0.03, respectively). And no significant difference was found in the circulating vaspin levels between the patients with MAFLD with a BMI ≥ 30 kg/m^2^ and the healthy controls (SMD = − 0.56 [− 1.33, 0.21], *P* < 0.00001). High heterogeneity (*P* = 0.002, *I*^*2*^ = 80%; *P* = 0.03, *I*^*2*^ = 68%; *P* < 0.00001, *I*^*2*^ = 93%, respectively) still existed and a random-effects model was selected again.

### Subgroup analysis by HOMA-IR

**Fig. 7:**
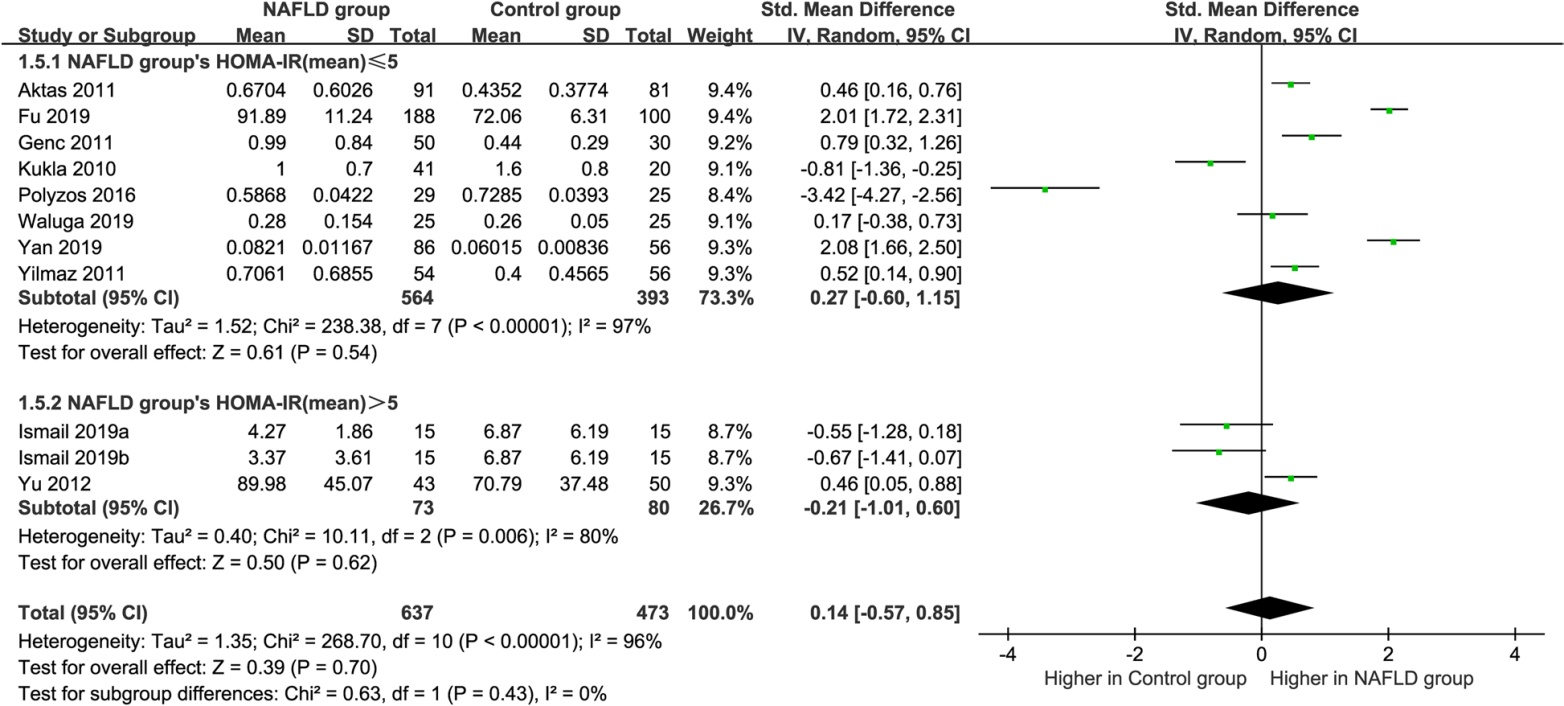
Forest plot of circulating vaspin levels between MAFLD and the healthy control group by HOMA-IR (Random-Effects Model, SMD)

Subgroup analysis by HOMA-IR chose a random-effects model owing to the high heterogeneity (HOMA-IR ≤ 5: *P* < 0.00001, *I*^*2*^ = 97%; HOMA-IR > 5: *P* = 0.006, *I*^*2*^ = 80%) (Fig. 7). The MAFLD group with HOMA-IR ≤ 5 and HOMA-IR > 5 had circulating vaspin levels that were not significantly different from those in healthy group (SMD = 0.27 [− 0.60, 1.15] and SMD = − 0.21 [− 1.01, 0.60], respectively).

### Subgroup analysis by severity

**Fig. 8:**
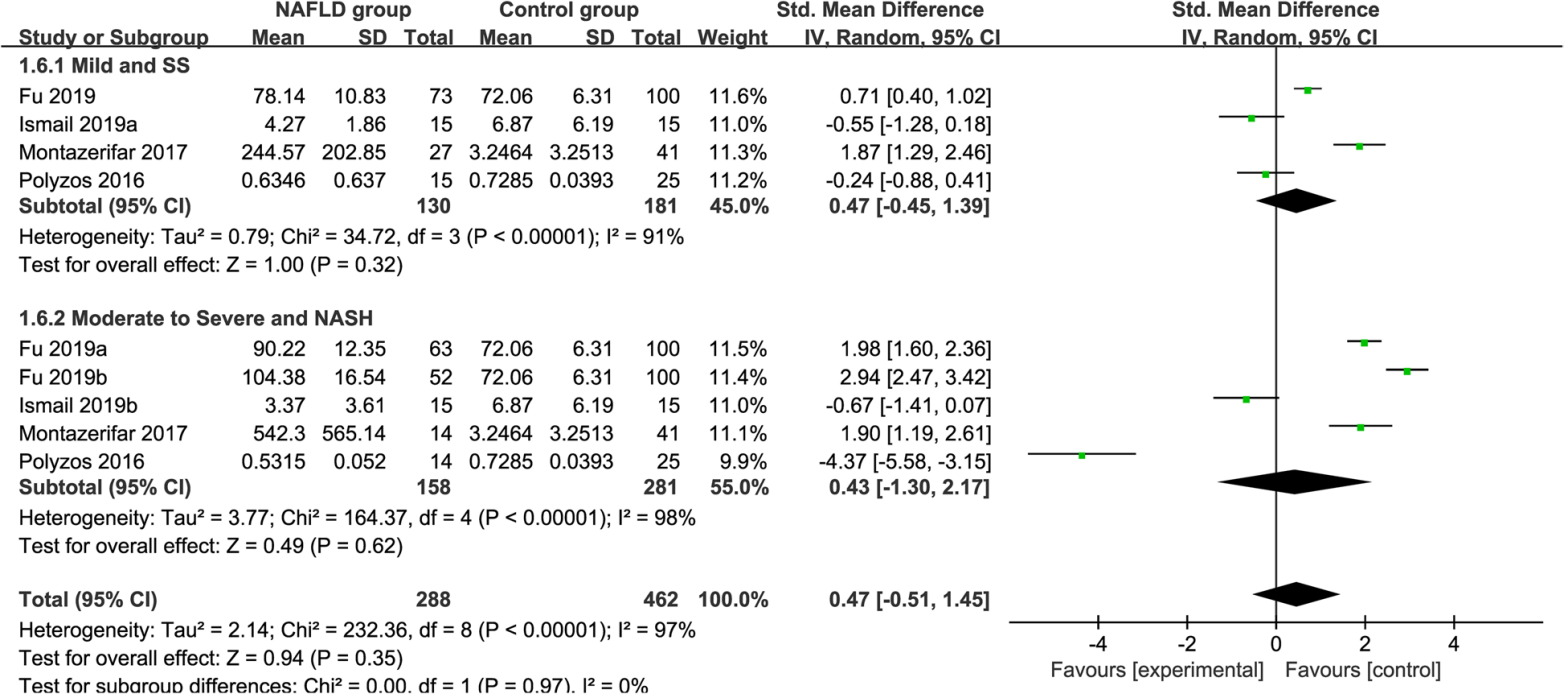
Forest plot of circulating vaspin levels between MAFLD and the healthy control group by severity (Random-Effects Model, SMD)

Subgroup analysis by severity used the random-effects model shown in Fig. 8. The circulating vaspin levels in the mild MAFLD + SS and moderate to severe MAFLD + NASH groups were not significantly different from those in the healthy individuals (SMD = 0.47 [− 0.45, 1.39]; SMD = 0.43 [− 1.30, 2.17]; respectively). High heterogeneity could not be neglected in this analysis (*P* < 0.00001, *I*^*2*^ = 97%).

### Subgroup analysis by NOS score

**Fig. 9:**
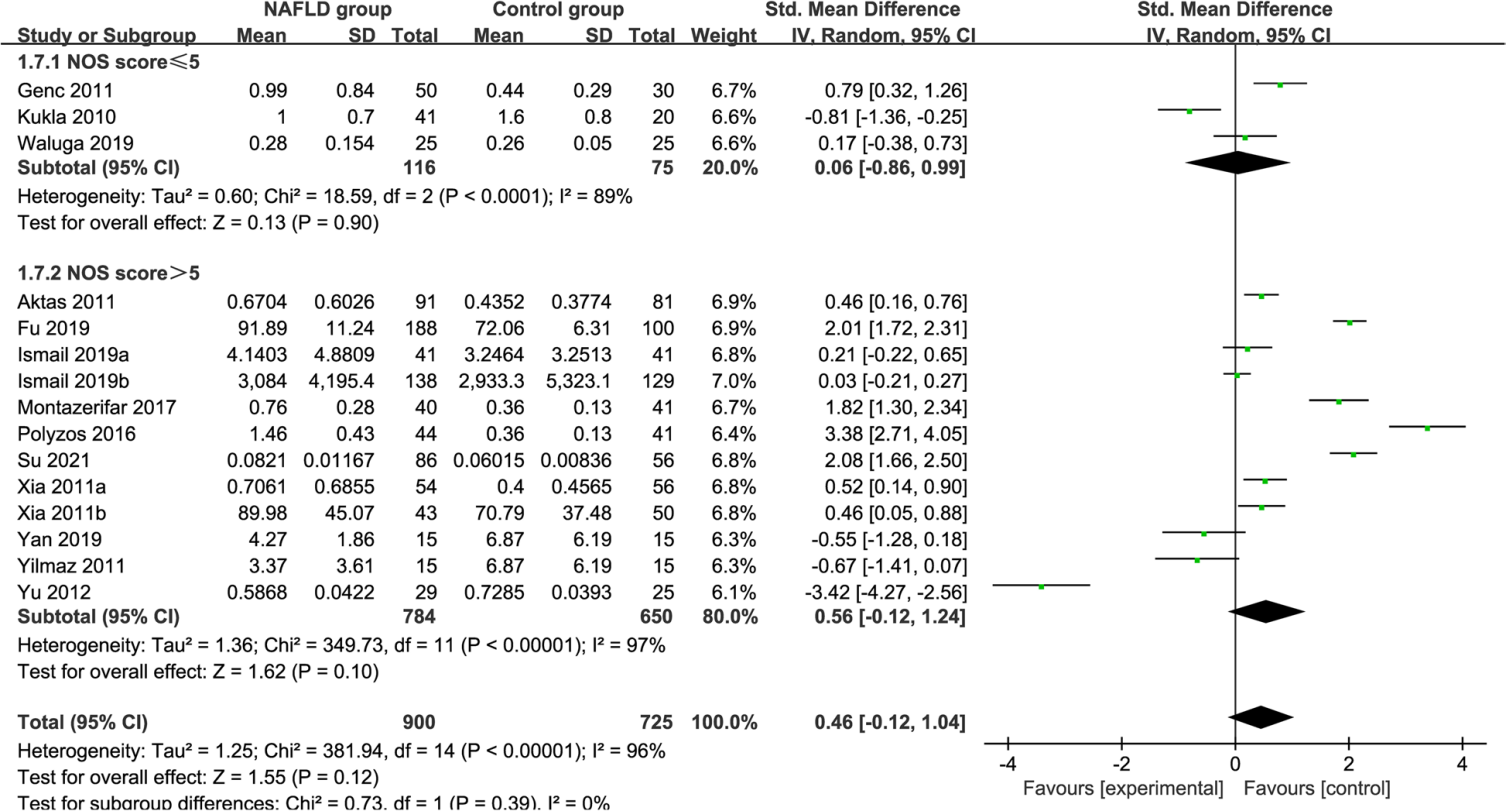
Forest plot of circulating vaspin levels between MAFLD and the healthy control group by NOS score

The NOS score was used as the basis of the subgroup analysis (Fig. 9). The random-effects model was selected as a result of the high heterogeneity (*P* < 0.00001, *I*^*2*^ = 96%). The circulating vaspin levels of the MAFLD patients with NOS score ≤ 5 and NOS score > 5 were not significantly different from those of the healthy controls (SMD = 0.06 [− 0.86, 0.99]; SMD = 0.56 [− 0.12, 1.24]; respectively).

### Meta-regression

A meta-regression analysis was performed for further exploration (Table 3). The univariate meta-regression demonstrated that BMI and area may be the sources of the heterogeneity. Age, HOMA-IR, and the NOS score may not be the sources of the heterogeneity (age: *P* = 0.545; BMI: *P* = 0.739; NOS score: *P* = 0.418). The multivariate meta-regression results for area and BMI demonstrated that BMI may better explain the existance of high heterogeneity (*P* = 0.032). The figures of meta-regression for all analyses are shown in Additional file 5.

**Table 3:** Meta-regression of the circulating vaspin levels and MAFLD

Covariates	No. Groups	Coefficient	Standard error	t	***P***	95%CI
Univariate meta-regression analysis						
Area	15	9.171	5.979	3.40	0.005	[2.243,37.504]
Age	15	1.030	0.048	0.62	0.545	[0.930,1.139]
BMI	16	0.710	0.052	−4.63	0.000	[0.606,0.832]
HOMA-IR	12	0.945	0.154	−0.34	0.739	[0.656,1.362]
NOS score	15	0.499	0.415	−0.84	0.418	[0.083,3.003]
Multivariate meta-regression analysis						
Area	16	2.776	2.277	1.24	0.235	[0.472,16.335]
BMI	16	0.780	0.081	−2.40	0.032	[0.623,0.976]
Cons	16	949.767	3246.888	2.01	0.066	[0.589,1,531,388]

*CI* Confidence interval, *BMI* Body mass index, *HOMA-IR* Homeostasis model assessment of insulin resistance, *NOS* Newcastle–Ottawa Scale

### Sensitivity analysis

**Fig. 10:**
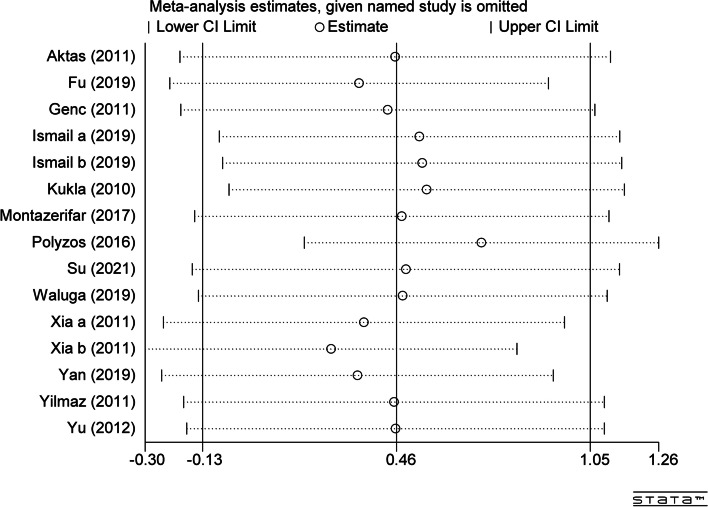
Sensitivity analysis plot of circulating vaspin levels between MAFLD and the healthy control group

The method of sensitivity analysis is to remove each single study individually (Fig. 10). The results suggest that no article had a significant impact on the results, which also indicates the stability of this study.

### Publication bias

**Fig. 11:**
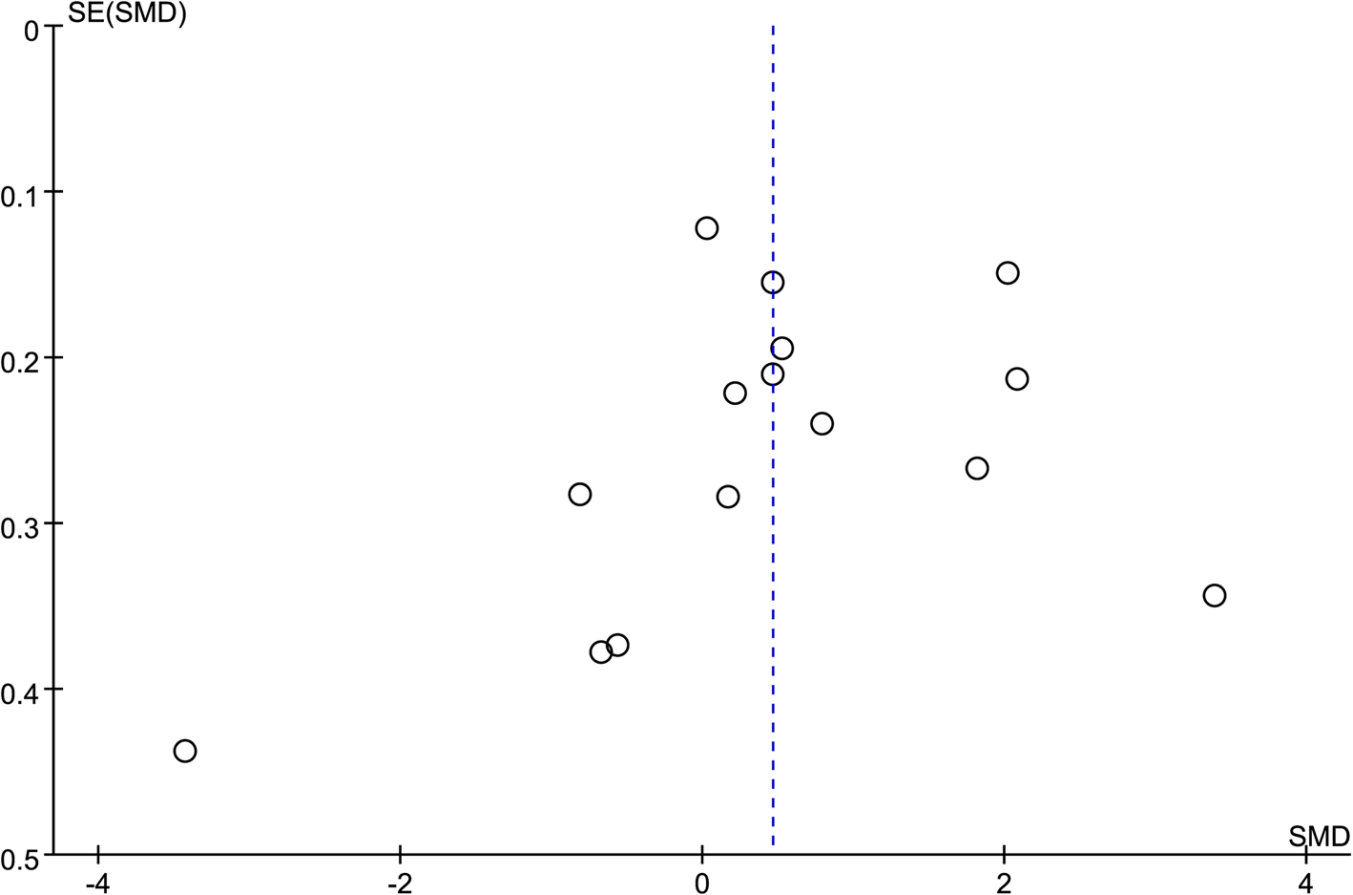
Funnel plot of circulating vaspin levels between MAFLD and the healthy control group

Egger’s and Begg’s tests demonstrated no significant differences in any of the comparisions (*P* > 0.05), indicating a low probability of publication bias (see Additional file 6). The funnel plot also demonstrated no significant publication bias (Fig. 11).

## Discussion

The diagnosis of MAFLD demands evidence of liver steatosis and the secondary causes of liver fatty accumulation should be excluded [38], such as excessive drinking. Pathophysiology suggests [39, 40] that it may be hepatic necrotising inflammation due to hepatic steatosis, lipotoxicity, and insulin resistance, which are closely linked to the degree of obesity and nutritional status of the body, emphasising the characteristics of metabolic abnormalities. Vaspin has 40.5% homology with α1-antitrypsin [41] and may play a compensatory role in metabolic disorders by mediating protease [42]. Liu et al. [43] speculated that vaspin inhibits inflammatory processes to improve insulin resistance by reducing the expression levels of inflammatory adipokines and vascular cell-derived cytokines. Meanwhile, some studies have pointed out that serine protease inhibitors, which are altered in structure or affect secretion, can lead to liver cirrhosis [44] and serum vaspin is associated with hepatocyte balloon degeneration [45]. There is a correlation between vaspin and liver diseases, especially with respect to cell degeneration and insulin resistance in the pathogenesis of MAFLD. Similarly, several studies have focused on the correlation between vaspin levels and other metabolic syndromes. Feng et al. [46] concluded that serum vaspin levels were highly expressed in obese subjects and T2DM patients compared with healthy controls. A meta-analysis by Mehrabani et al. [47] including 77 case-control studies with 8239 participants reported that the serum vaspin levels in polycystic ovarian syndrome (PCOS) patients were significantly higher than in controls. While Lin et al. [48] revealed that no significant difference was found between non-obese patients with PCOS and non-obese healthy group in circulating vaspin levels. However, no meta-analysis has analysed the correlation between the circulating vaspin levels and MAFLD. Therefore, determining the possible relationship between vaspin and MAFLD and the influence of different factors on their correlation will also have certain guiding significance and value for clinical and scientific research.

The present meta-analysis included 13 studies with 900 cases and 669 controls, including patients from China, Turkey, Poland, Iran, Greece, and Egypt. The pooled results showed the circulating vaspin levels in MAFLD patients had not significant difference compared with those in healthy controls, with high heterogeneity. Subgroup analysis confirmed that area and BMI could be considered as the sources of the high heterogeneity, and univariate meta-regression further verified this. Age, HOMA-IR, and the NOS score may not be sources of heterogeneity. Multivariate meta-regression analysis revealed BMI was the only source of heterogeneity. Geographically, the MAFLD group in Asia had higher vaspin levels than those in health individuals, whereas no significant difference existed between the MAFLD patients in other areas and the healthy individuals. In addition to ethnic differences, the insufficient number of studies in other areas may also contribute to this outcome. When analysed based on BMI, the MAFLD groups with BMI < 25 kg/m^2^ and 25 kg/m^2^ ≤ BMI < 30 kg/m^2^ had significantly higher vaspin levels. In contrast, significant difference did not exist in the vaspin levels between the MAFLD patients with BMI ≥ 30 kg/m^2^ and the healthy group. It was observed that vaspin levels in MAFLD patients with a normal body size or between overweight and obesity were higher than those in the healthy controls, and the differences between MAFLD with a normal body size and the healthy controls were even more pronounced. Similarly, Loeffelholz et al. [49] observed an interaction between the BMI, sex, and vaspin in lean and normal-weight subjects (BMI < 25 kg/m^2^), while no such association was discovered in overweight individuals. As vaspin is mainly produced from visceral adipose tissue, it can be expected there are associations between vaspin and BMI or body fat percentage, and may be positively correlated before reaching a certain threshold. Differences in circulating vaspin levels in MAFLD patients with different BMI may be the focus of future research. Furthermore, obesity may be an important cause of metabolic syndrome and further rise the risk of cardiovascular disorders [50]. Assuming that the specificity of vaspin levels in nonobese patients is confirmed, it might be possible to distinguish patients from normal-sized people at an earlier stage to effectively prevent other related diseases.

### Strengths and limitations

As far as team can find, this meta-analysis is the first one to focus on the correlation between circulating vaspin levels and MAFLD. Subgroup, univariate, and multivariate meta-regression analysis were applied to identify BMI as the source of heterogeneity, and a detailed explanation and discussion were made around it. In addition, Egger’s test, Begg’s test, sensitivity analysis, and funnel plot all confirmed the robustness of the pooled results. However, there are several limitations could not be neglected in this meta-analysis. First, the number of articles included in this study was insufficient, which may be slightly insufficient in representativeness. Additionally, most articles come from Asia and have certain regional tendencies. Second, there were significant differences between the values in each study, even though the indicator of circulating vaspin levels was converted into the same units. The maximum is 91.89 ng/mL and the minimum is 0.05 ng/mL. This may be associated with the different detection methods used in different studies and the different experimental kits from different manufacturers. Third, MAFLD can usually be divided into mild, moderate, and severe, and can also be divided into SS and NASH, according to pathology. Many other similar studies have suggested a link between biological indicators and severity, but the results of this study are not consistent. This may be due to insufficient relevant subgroup data. Finally, because the indicators involved in each study were different, the study only selected the common indicators: area, age, BMI, HOMA-IR, and severity. It is believed that there must be other factors affecting heterogeneity, such as sex, while the included studies related to vaspin and MAFLD have provided limited data. More research is needed to address these points.

## Conclusion

In summary, this meta-analysis revealed no significant difference was observed between MAFLD patients and healthy people in circulating vaspin levels. However, in Asia, the circulating vaspin levels were significantly higher in MAFLD group than healthy individuals, with high heterogeneity. MAFLD group with BMI < 25 kg/m^2^ and 25 kg/m^2^ ≤ BMI < 30 kg/m^2^ had significantly higher vaspin levels than those in the health. Subgroup and univariate meta-regression analysis indicated that BMI and area could be the sources of heterogeneity. Further multivariate meta-regression verified that BMI could be the soruce of heterogeneity. Owing to the limited number of included articles, it can only provide a reference for the correlation between vaspin and MAFLD and promote new ideas for clinical and scientific research. The characteristics of vaspin levels in non-obese patients with NAFLD might be worth exploring for clinical application. In the immediate future, multi-centre, multi-regional, and multi-faceted research is still an important premise to draw more comprehensive and perfect conclusions.

## Supplementary Information

**Additional file 1.** PROSPERO: Number CRD42022301367.

**Additional file 2.** PRISMA 2009 checklist.

**Additional file 3.** Databases retrieval strategy.

**Additional file 4.** The results of GRADE system.

**Additional file 5.** Figures of meta-regression for all analysis.

**Additional file 6.** Figures of Egger’s test and Begg’s test.

## Data Availability

All data generated or analysed during this study are included in this published article and its supplementary information files.

## Abbreviations

MAFLD: Metabolic Associated Fatty Liver Disease
NAFLD: Nonalcoholic Fatty Liver Disease
SS: Simple Steatosis
NAFL: Nonalcoholic Fatty Liver
NASH: Nonalcoholic Steatohepatitis
T2DM: Type 2 Diabetes Mellitus
SMD: Standardized Mean Difference
CI: Confidence Intervals
NOS: Newcastle-Ottawa Scale
WHO: World Health Organization
BMI: Body Mass Index
HOMA-IR: Homeostasis Model Assessment of Insulin Resistance
