# Supplementary material for: Association of circulating vaspin levels and patients with metabolic-associated fatty liver disease: a systematic review and meta-analysis
Source: Lipids Health Dis. 2022 Jul 2;21:57. doi: 10.1186/s12944-022-01658-2 (PMC9250748; doi:10.1186/s12944-022-01658-2)
Supplement: Supplementary file 1 — Additional file 1. PROSPERO: Number CRD42022301367. [file 12944_2022_1658_MOESM1_ESM.pdf]

To enable PROSPERO to focus on COVID-19 submissions, this registration record has undergone basic automated checks for eligibility and is published exactly as submitted. PROSPERO has never provided peer review, and usual checking by the PROSPERO team does not endorse content. Therefore, automatically published records should be treated as any other PROSPERO registration. Further detail is provided here.

Citation

Yuqing Zhu, Yani Ke, Shuaihang Chen. Association of circulating Vaspin levels in patients with Nonalcoholic Fatty Liver disease(NAFLD): a systematic review and meta-analysis.. PROSPERO 2022 CRD42022301367 Available from: https://www.crd.york.ac.uk/prospero/display_record.php?ID=CRD42022301367

Review question

1) To explore the difference of circulating Vaspin level between NAFLD patients and healthy people.

2) The value of Vaspin in predicting NAFLD occurrence.

Our systematic review and meta-analysis aim to combing existing relevant literature, draw reliable and accurate conclusions.

Searches

We will search some databases including PubMed, Cochrane Library, Cochrane Register of Controlled

Trials(CENTRAL), EMBASE, CNKI, WANFANG and CBM, be using the terms “Vaspin" AND "Nonalcoholic fatty liver disease" OR "NAFLD" OR "nonalcoholic steatohepatitides" OR "NASH" OR "Nonalcoholic fatty liver".

Furthermore, reference lists of enrolled studies and publications with citations of included papers will also be reviewed for suitable papers. We will also contact study authors by e-mail to clarify additional studies and ask for the missing data.

Types of study to be included

Case-control studies or cohort studies will be included.

Condition or domain being studied

Adipose tissue, especially visceral adipose tissue, plays an important role in the pathogenesis of NAFLD. As one of the adipokines derived from visceral adipose tissue, Vaspin participates in the progression of NAFLD disease through insulin resistance, hepatic steatosis, liver inflammation and liver fibrosis, Which is a new target for alleviating obesity and reducing insulin resistance.

Participants/population

Inclusion Criteria:

1) Enrolled patients should be explicitly diagnosed with nonalcoholic fatty liver disease, and the control group should be health.

2) All patients should be adults over 18 years old.

3) Patients should not receive any intervention prior to monitoring data.

Exclusion Criteria:

1) Patients who had other severe active comorbidities.

Intervention(s), exposure(s)
Diagnosed with NAFLD (Definitive diagnosis is required using internationally accepted diagnostic criteria);

Circulating Vaspin level (Enzyme-linked immunosorbent assay was used).

Comparator(s)/control
In cohort studies, high-exposure group is NAFLD patients, and the low-exposure group is health.

In case-control studies, NAFLD patients are case group, health are control group.

Main outcome(s)
1) Circulating Vaspin level.

2) Diagnosed analysis data, including sensitivity, spectivity, PLR, NIR.

Additional outcome(s)
CRP, IL-6, TC, TG, LDL-C, HDL-C, ALT, and AST, etc.

Data extraction (selection and coding)
Study selection: The number of researchers complies with the standard. Two researchers independently review the literature and cross check it. In case of any divergence, they will discuss it or is submitted to a third party for decision.

Data extraction: The number of researchers complies with the standard. Two researchers independently extract the literature data and cross check them. In case of any divergence of literature, they will discuss it or is submitted to a third party for decision.

Risk of bias (quality) assessment

According to Newcastle-Ottawa Scale (NOS) scale, the quality of the proposed studies was assessed, which involved three dimensions including selection, comparability, and exposure.

Strategy for data synthesis
Review manager 5.2 and Stata 15 software will be used for statistical analysis to perform meta-analysis. Heterogeneity will be checked by the X^2 test and the I^2 statistic.

The criteria for identification of heterogeneity will be a P value less than 0.10 for the X^2 test and an I^2 statistic greater than 50%.

Analysis of subgroups or subsets
Subgroup analysis was used when the statistical results were heterogeneous or could not be combined, which may be performed by age, gender, country, and laboratory examination index.

Contact details for further information
YuQing Zhu
zhuyuqingnb@163.com

Organisational affiliation of the review
Zhejiang Chinese Medical University (ZCMU)
https://www.zcmu.edu.cn/

Review team members and their organisational affiliations
Yuqing Zhu. Zhejiang Chinese Medical University
Yani Ke. Zhejiang Chinese Medical University
Shuaihang Chen. Zhejiang Chinese Medical University

Type and method of review

????, ????

Anticipated or actual start date
12 February 2022

Anticipated completion date
01 May 2022

Funding sources/sponsors
Zhejiang Chinese Medical University.

Zhejiang Provincial Hospital of Traditional Chinese Medicine.

Conflicts of interest

Language
English

Country
China

Stage of review
Review Ongoing

Subject index terms status
Subject indexing assigned by CRD

Subject index terms
MeSH headings have not been applied to this record

Date of registration in PROSPERO
09 February 2022

Date of first submission
09 January 2022

Stage of review at time of this submission
The review has not started

**Stage**	**Started**	**Completed**
Preliminary searches	No	No
Piloting of the study selection process	No	No
Formal screening of search results against eligibility criteria	No	No
Data extraction	No	No
Risk of bias (quality) assessment	No	No
Data analysis	No	No

The record owner confirms that the information they have supplied for this submission is accurate and complete and they understand that deliberate provision of inaccurate information or omission of data may be construed as scientific misconduct.

The record owner confirms that they will update the status of the review when it is completed and will add

publication details in due course.

Versions

09 February 2022
09 February 2022
