# Supplementary material for: Association of circulating vaspin levels and patients with metabolic-associated fatty liver disease: a systematic review and meta-analysis
Source: Lipids Health Dis. 2022 Jul 2;21:57. doi: 10.1186/s12944-022-01658-2 (PMC9250748; doi:10.1186/s12944-022-01658-2)
Supplement: Supplementary file 3 — Additional file 3. Databases retrieval strategy. [file 12944_2022_1658_MOESM3_ESM.pdf]

Databases	Search terms	Number of records
Pubmed	Search : (((((('vaspin') OR ('vaspin protein')) OR ('Serine Proteinase Inhibitors'[MeSH Terms])) OR ('Serpins'[MeSH Terms])) OR ('SERPINA12 protein, human' [Supplementary Concept])) AND ((((((((((((('nonalcoholic steatohepatitides') OR ('nonalcoholic steatohepatitis')) OR ('nonalcoholic fatty liver disease')) OR ('nafld')) OR ('non alcoholic fatty liver disease')) OR ('nash')) OR ('steatohepatitides, nonalcoholic')) OR ('steatohepatitis, nonalcoholic')) OR ('liver, nonalcoholic fatty')) OR ('fatty liver')) OR ('nonalcoholic fatty liver')) OR ('metabolic associated fatty liver disease')) OR ('mafld')) OR ('nonalcoholic fatty liver'[MeSH Terms]))	310
Cochrane Library	#1MeSH descriptor:[Non-alcoholic Fatty Liver Disease] explode all trees<br>#2'fatty liver'<br>#3'liver, nonalcoholic fatty'<br>#4'steatohepatitides, nonalcoholic'<br>#5'steatohepatitis, nonalcoholic'<br>#6'nash'<br>#7'non alcoholic fatty liver disease'<br>#8'nafld'<br>#9'nonalcoholic fatty liver'<br>#10'nonalcoholic fatty liver disease'<br>#11'nonalcoholic steatohepatitis'<br>#12'nonalcoholic steatohepatitides'<br>#13'metabolic associated fatty liver disease'<br>#14'mafld'<br>#15 #1 or #2 or #3 or #4 or #5 or #6 or #7 or #8 or #9 or #10 or #11 or #12 or #13 or #14<br>#16'vaspin'<br>#17'vaspin protein'<br>#18MeSH descriptor:[Serpins] explode all trees<br>#19MeSH descriptor:[Serine Proteinase Inhibitors] explode all trees<br>#20'serpinA12'<br>#21 #16 or #17 or #18 or #19 or #20<br>#15 and #21	11
EMBASE	#1'nonalcoholic fatty liver'/exp<br>#2'fatty liver'<br>#3'liver, nonalcoholic fatty'<br>#4'steatohepatitides, nonalcoholic'<br>#5'nonalcoholic steatohepatitis'<br>#6'nash'<br>#7'non alcoholic fatty liver disease'<br>#8'nafld'	1064

	#9'nonalcoholic fatty liver disease'<br>#10'nonalcoholic fatty liver'<br>#11'nonalcoholic steatohepatitis'<br>#12'nonalcoholic steatohepatitides'<br>#13'metabolic associated fatty liver disease'<br>#14'mafld'<br>#15 #1 OR #2 OR #3 OR #4 OR #5 OR #6 OR #7 OR #8 OR #9 OR #10 OR #11 OR #12 OR #13 OR #14<br>#16'vaspin'/exp<br>#17'vaspin protein'<br>#18'serine proteinase inhibitor'/exp<br>#19'serpins'<br>#20'serpina12 protein human'/exp<br>#21'serpinA12'<br>#22 #16 OR #17 OR #19 OR #20 OR #21<br>#15 AND #22	
CNKI	(SU =非酒精性脂肪肝 OR SU =非乙醇性脂肪肝 OR SU =非酒精性脂肪性肝病 OR SU =非酒精性单纯性脂肪肝 OR SU =非酒精性脂肪性肝炎 OR SU =非酒精性肝炎 OR SU =脂肪性肝炎 OR SU =代谢相关性脂肪肝 OR SU =代谢相关性脂肪肝病) AND (SU =丝氨酸蛋白酶抑制剂 OR SU =vaspin OR SU =serpinA12 OR SU =serpins)	22
Wanfang	全部：(非酒精性脂肪肝 or 非乙醇性脂肪肝 or 非酒精性脂肪性肝病 or 非酒精性单纯性脂肪肝 or 非酒精性脂肪性肝炎 or 非酒精性肝炎 or 脂肪性肝炎 or 代谢相关性脂肪肝 or 代谢相关性脂肪肝病) and (丝氨酸蛋白酶抑制剂 or vaspin or serpinA12 or serpins)	2
CBM	("非酒精性脂肪肝"[全部字段] OR "非乙醇性脂肪肝"[全部字段] OR "非酒精性脂肪性肝病"[全部字段] OR "非酒精性单纯性脂肪肝"[全部字段] OR "非酒精性脂肪性肝炎"[全部字段] OR "脂肪性肝炎"[全部字段] OR "非酒精性肝炎"[全部字段] OR "代谢相关性脂肪肝"[全部字段] OR "代谢相关性脂肪肝病"[全部字段]) AND ("丝氨酸蛋白酶抑制剂"[全部字段] OR "vaspin"[全部字段] OR "serpinA12"[全部字段] OR "serpins"[全部字段])	23
