# Supplementary material for: Association of circulating vaspin levels and patients with metabolic-associated fatty liver disease: a systematic review and meta-analysis
Source: Lipids Health Dis. 2022 Jul 2;21:57. doi: 10.1186/s12944-022-01658-2 (PMC9250748; doi:10.1186/s12944-022-01658-2)
Supplement: Supplementary file 4 — Additional file 4. The results of GRADE system. [file 12944_2022_1658_MOESM4_ESM.pdf]

GRADE summary of findings table.

Outcome	Participants (studies)	Risk of bias	Inconsistency	Indirectness	Imprecision	Other considerations	Overall certainty of evidence
Vaspin	13 observational studies	Serious[1]	Serious[2]	Not serious	Not serious	Plausible confounding[3]	VERY LOW

[1]Serious risk of bias due to the NOS score (5-6)
[2]Serious inconsistency due to significant heterogeneity ($I^2 > 50\%$)
[3]all plausible residual confounding would reduce the demonstrated effect. Some of the studies do not match the basic information of two groups, like age, BMI and so on.
