# Supplementary figures and images for: Association of circulating vaspin levels and patients with metabolic-associated fatty liver disease: a systematic review and meta-analysis
Source: Lipids Health Dis. 2022 Jul 2;21:57. doi: 10.1186/s12944-022-01658-2 (PMC9250748; doi:10.1186/s12944-022-01658-2)

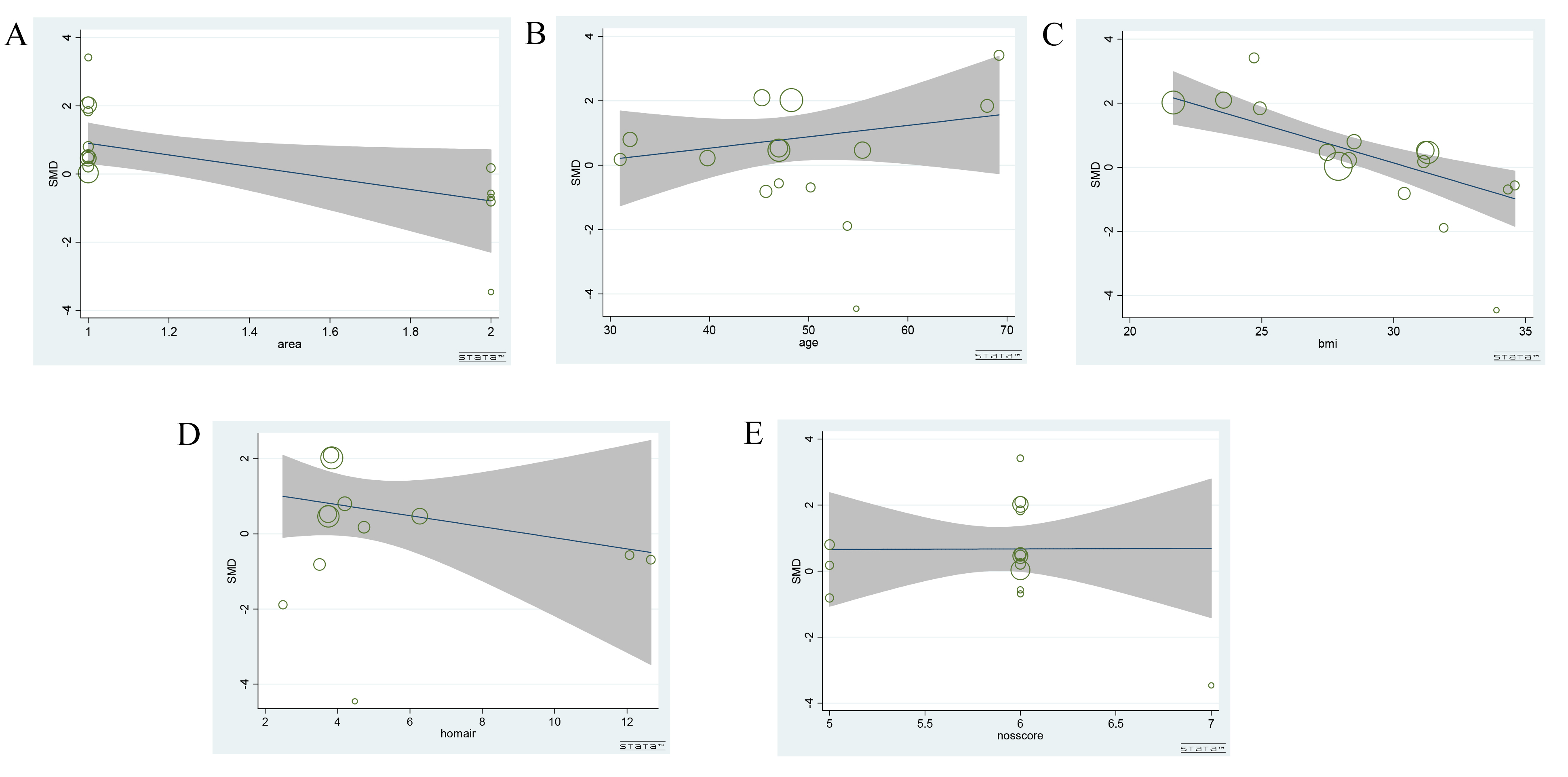

Supplement: Supplementary file 5 — Additional file 5. Figures of meta-regression for all analysis. [file 12944_2022_1658_MOESM5_ESM.jpg]

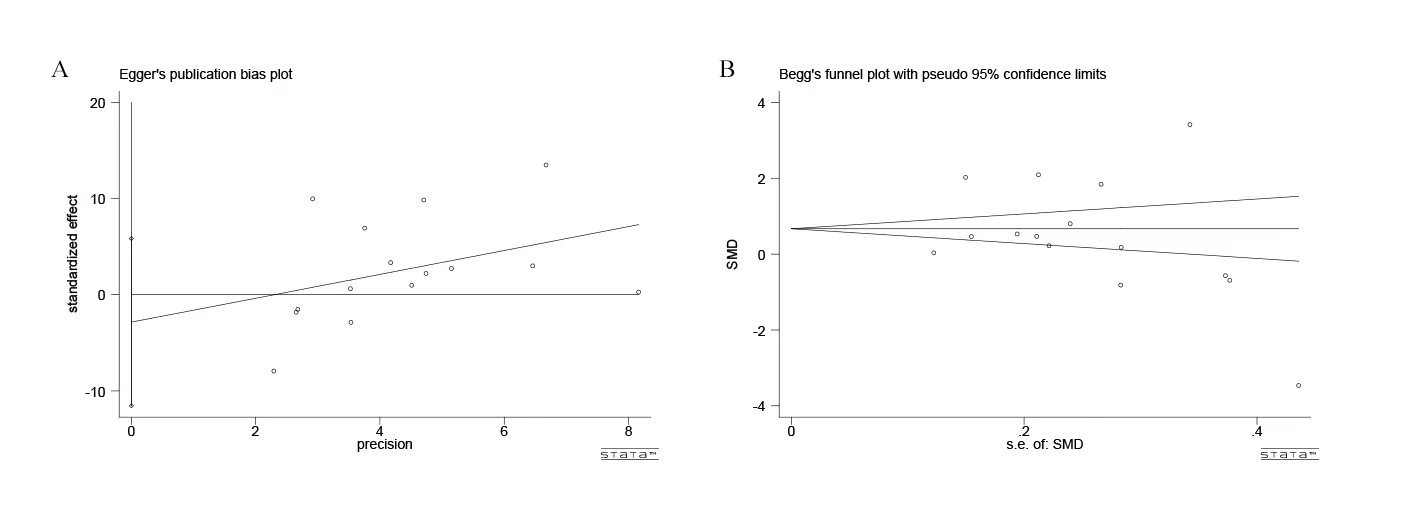

Supplement: Supplementary file 6 — Additional file 6. Figures of Egger’s test and Begg’s test. [file 12944_2022_1658_MOESM6_ESM.jpg]
